# National survey of Canadian residents and program directors regarding parental leave during residency

**DOI:** 10.36834/cmej.68486

**Published:** 2020-09-23

**Authors:** Karen Willoughby, Marie Julien, Benjamin Rich Zendel, Vernon Curran

**Affiliations:** 1 Faculty of Medicine, Memorial University, Newfoundland and Labrador, Canada; 2 Université Laval, Quebec, Canada; 3 Aging Research Centre, Grenfell Campus, Memorial University, Newfoundland and Labrador, Canada

## Abstract

**Background:**

Despite the advantages of having a child as a medical resident, the transition back to residency after parental leave can be challenging. This study is the first to investigate this issue using a nation-wide Canadian sample of both residents and program directors.

**Method:**

A questionnaire was developed and made available online. Respondents included 437 female residents, 33 male residents, and 172 residency program directors. The mean length of parental leave was nine months for female residents and six weeks for male residents. Almost all female residents (97.5%) breastfed with an average duration of 12 months. The top challenges reported by residents were feeling guilty for being away from their family, long and unpredictable work hours, sleep deprivation, and finding time to study. When female residents and program directors were matched to both school and program (*N* = 99 pairs), there was no difference in the total number of challenges reported, but program directors reported offering significantly more accommodations than female residents reported being offered, *t(196)* = 13.06, *p* < .001.

**Results:**

Our data indicate there is a need for better communication between resident parents and program directors, as well as clear program-specific parental leave policies, particularly for supporting breastfeeding mothers as they return to work.

## Abstract (fr)

**Contexte:**

Malgré les avantages d’avoir un enfant au cours d’une résidence en médecine, le retour à la résidence à la suite d’un congé parental peut s’avérer difficile. Cette recherche est la première qui étudie cette question en utilisant un échantillon national comptant à la fois des résidents et directeurs de programmes canadiens.

**Méthode:**

Un questionnaire a été préparé et affiché en ligne. Parmi les personnes interrogées, on comptait 437 résidentes, 33 résidents et 172 directeurs et directrices de programmes de résidence. La durée moyenne des congés parentaux était de neuf mois pour les résidentes et six semaines pour les résidents. Presque toutes les résidentes (97,5%) ont allaité en moyenne pendant douze (12) mois. Les plus grands défis que les résidents ont signalés étaient la culpabilité de ne pas être avec leur famille, les longues et imprévisibles heures de travail, le manque de sommeil et la difficulté à trouver le temps pour étudier. Lorsque les résidentes et directeurs et directrices de programme étaient jumelés à l’école et au programme (*N* = 99 paires), le nombre total de difficultés rapportées est demeuré inchangé. Cependant, les directeurs et directrices de programme ont signalé qu’ils offraient beaucoup plus d’accommodements que ce que les résidentes ont déclaré qu’on leur proposait, *t(196)* = 13.06, *p* < .001.

**Résultats:**

Nos données indiquent qu’il est nécessaire d’améliorer la communication entre les résidents parents et les directeurs de programme ainsi que les politiques qui régissent les programmes de congés parentaux, notamment pour aider les mères qui allaitent au moment de retourner au travail.

## Introduction

An increasing number of medical residents across North America are having children during residency.^1^ Within Canada, this change may be associated with higher rates of females than males enrolled in Canadian medical schools in recent years (e.g., from 35% in 1980 to 56% in 2017).^2^ Other reasons may include being older when starting medical school and the opportunity to receive federal parental leave funding as a medical resident.^3^^,^^4^ Although residency may be an optimal time to have a child due to financial support and protected time for parental leave, numerous studies have found that many resident parents struggle with numerous challenges associated with taking parental leave during residency (see Finch, 2003,^1^ and Humphries et al., 2017,^5^ for a review).^6^^-^^11^

Within Canada, studies investigating the challenges of having children during residency have been limited to surveys of residents in either a specific residency program, such as Obstetrics and Gynecology,^10^^,^^12^ General Surgery,^11^ Psychiatry,^13^ and Family Medicine,^14^ or a specific province, such as Québec.^15^^,^^16^ One of the first qualitative studies to explore the struggles associated with taking a parental leave during residency in Canada showed that female family medicine residents felt their greatest challenges occurred in the first few months of returning to residency. These residents reported receiving much less support during this period than they did while pregnant.^14^

The most common challenges reported by female residents when returning to residency after taking parental leave included fatigue from sleep deprivation, challenges related to breastfeeding or pumping at work, childcare difficulties, and lack of support from partners and/or preceptors.^8^^,^^9^^,^^14^^,^^17^ These appear to be exacerbated by the fact that many program directors report receiving no guidance or formal policies for how to support resident parents as they return from parental leave.^11^^,^^18^ For instance, most institutional or residency association parental leave policies only cover basic human rights issues related to pregnancy, the length of parental leave, and in some cases breastfeeding.^11^ A recent review of all Canadian residency program parental leave policies noted that support for resident parents varies considerably across institutions and residency programs and does not appear to be consistent, universal, or guaranteed.^3^ As a result, support for resident parents appears to be dependent on how parental leave is approached and handled by each program director, instead of on institutional policies.^3^^,^^5^ Several reports have outlined specific accommodations that could help support residents as they return from parental leave, such as the option to return to residency part-time, to remain involved in academic and/or clinical work during parental leave, the provision of on-site childcare, as well as the provision of time, privacy, and storage to help maintain breastfeeding.^1^^,^^4^^,^^11^^,^^14^ However, no study has formally examined whether Canadian resident parents are being offered any of these accommodations on a national scale.

Although it is important to develop parental leave policies that are unique to each specialty, a nationwide study from all specialties can provide important data that can be used by various stakeholders (e.g., program directors, residency preceptors, residency associations) to help create or improve institutional program-specific parental leave policies. Collecting these data, along with other pertinent data for prospective resident parents (e.g., why previous residents chose to have a child during residency, when they informed their program director of the pregnancy, etc.) can also serve to better inform and prepare future resident parents for the challenges that lie ahead. Gathering information from both residents and program directors is important to assess whether program directors are aware of the difficulties resident parents face and to provide more complete and accurate information about what accommodations are currently being offered to resident parents from multiple perspectives. Our study is unique in three respects. This is likely the first national study to examine common issues associated with parental leave by surveying both residents and program directors from all medical schools and residency programs in Canada. We also recruited a large sample of physician mothers who were either current residents or had completed a residency within the last 20 years. This range allowed us to examine whether the experiences of resident mothers have changed over time. Finally, this study is one of only a few studies to survey male resident parents, who also face challenges when returning to residency after the birth of their child that may often be overlooked.

## Methods

For our study, we used quantitative surveys to collect a wide range of data from a large number of participants in a short time period. To develop our resident and program director surveys, we conducted a literature review using PubMed database using multiple search terms (e.g., residency, medical training, postgraduate medical education, parental leave, pregnancy) that included articles published between Jan, 1, 1970 and November 1, 2018 with data from North America. Articles were screened by title and abstract for relevancy (e.g., having a focus on the transition back to residency after parental leave). We searched the references in pertinent research articles, meta-analyses, and commentaries for any studies not discovered in the PubMed search. We adapted the resident and program director surveys from Hutchinson et al.’s^8^ previously piloted and published surveys (permission to use these surveys was obtained by contacting the lead author). We reviewed the results of several other quantitative and qualitative studies^9,^^10^^,^^12^^,^^14^^,^^16^ and developed additional questions based on these results that included extra demographic information,^10^^,^^16^ challenges associated with returning to residency after parental leave,^14^^,^^16^ requested accomodations,^16^ program directors’ attitudes towards having a child during residency, and satisfaction regarding parental leave experiences that served as valuable exploratory data from a nationwide sample. We also included questions regarding a few topics that would be informative for prospective resident parents (e.g., why residents chose to have a child in residency, when to inform program directors of a pregnancy, etc.). Participants were given an opportunity to provide open-ended comments at the end of the survey. We applied thematic analysis in analysing and summarizing these open-ended comments. The questionnaire for male residents was identical to the one for female residents with the exception that it excluded questions about breastfeeding. All questionnaires were also available in French and were translated by a native French speaker (copies of French questionnaires are available upon request). Most questions elicited a yes (1) or no (0) response; however, some questions had a five-point Likert scale (1 = *very satisfied*, 5 = *very dissatisfied*). The resident questionnaire was piloted with two individuals to ensure clarity of the survey questions and to estimate the time required to complete the survey. Since we derived our questions from previously published surveys and empirical literature, we assumed we had sufficient construct representation in our surveys. Other sources of validity evidence were not part of our study. See Supplemental data for copies of the questionnaires.

From November 2018 to April 2019, surveys were available online using Qualtrics. Invitations to participate in the study were sent to program directors by either contacting their postgraduate medical education department to distribute a letter of invitation or emailing them directly. Current and past residents were recruited by contacting provincial residency associations to advertise in their newsletters, and advertising on targeted social medial platforms (e.g., private Facebook groups specifically for physician mothers). Based on our data, we are able to estimate response rates for our study of approximately 13.6% for female residents who took parental leave in the last 10 years (i.e., 377 from our study, divided by 2765 as reported by program directors; see footnote for the calculation), 3.0% for male residents who took parental leave in the last 10 years (i.e., 31 from our study, divided by 1029; see footnote^1^), and 30.8% for current Program directors from programs in the CARMS R1 program list (i.e., 117 from our study divided by approximately 380 nationwide). A 30% response rate is typical for studies using online questionnaires that are able to directly contact the entire target population.^7^^,^^8^^,^^9^ The study was reviewed and approved by the Health Research Ethics Authority (HREA) at Memorial University of Newfoundland. Consent was implied when participants clicked ‘next page’ after reading the information page, and this was outlined in the information page description.

Four female residents were excluded from the study because they had not yet finished their maternity leave. Eleven program directors reported having no experience with a resident taking parental leave; thus, only their demographic data were included in the study. Due to the small number of male residents in the study (*N* = 33), data from these participants is presented for descriptive purposes only. Finally, to control for multiple comparisons, a significant level of *p* < .01 was used for all analyses.

## Results

### Demographic data

Participants included 437 female and 33 male past or current residents who took parental leave during a Canadian residency program, as well as 172 current Canadian residency program directors. Twenty female residents (4.6%), five male residents (15.2%), and 38 program directors (22.1%) completed the French version of the questionnaires. Demographic data for each group, including training setting and residency program are presented in Table 1 (See Appendix A). All 17 Canadian medical schools are represented in both the female resident and program director groups. The residency programs reported by participants were grouped according to the 2019 Canadian Resident Matching Service (CARMs) R1 program list (see https://www.carms.ca/program-descriptions/) with additional groupings for Medicine Subspecialties, Pediatric Subspecialties, and Enhanced Skills in Family Medicine.

### Why residents chose to have a child during residency

For most female (*N* = 416; 95.2%) and male (*N* = 32; 97.0%) residents, having a child during residency was planned. The top five reported reasons why female residents chose to have a child during residency were: i) advanced maternal age, ii) financial benefits, such as federal parental leave funding, provincial top-up funding and medical benefits, (iii) feeling ready to have a child, (iv) having the option to take a full year of parental leave, and v) because they felt it was easier to transition back to residency, due to the supervised learning environment, compared to taking parental leave after residency. For male residents, advanced parental age and being ready to have a child were the two main reasons reported for having a child during residency. When asked if they would recommend having a child during residency, the majority of female (*N* = 309; 70.7%) and male (*N* = 26; 78.8%) residents reported yes. Similarly, 148 (86.0%) program directors stated they would not discourage residents from having a child during residency, and many program directors commented that they felt residency was the best time to have children.

### When residents informed program directors about taking a parental leave

Most female (*N =* 292; 66.8%) and some male (*N =* 13; 39.4%) residents reported informing their program director that they were having a child between 12-20 weeks of gestation, while only a small percentage (22.9% of females and 15.2% of males) did so before 12 weeks of gestation. Similarly, 104 program directors (61.5%) reported they were typically informed about the intention to take parental leave between 12-20 weeks, while only 6.5% reported being informed before 12 weeks of gestation. One program director commented, “*I wish residents would tell me earlier so that I can plan things such as getting their rural rotations out of the way before the baby is born and avoiding heavy rotations late in gestation*.” The majority of female (*N =* 347; 79.4%) and male (*N =* 21; 81.8%) residents reported that their program director reacted either positively or very positively when they were informed about the pregnancy. Interestingly, there was a significant positive correlation between when female residents informed their program directors about their pregnancy and their perceived reaction from their program directors (*r* =.143; *p* = .003); more positive reactions were associated with earlier disclosure.

### Length of parental leave

The mean reported length of parental leave was approximately nine months for female residents and six weeks for male residents. For female residents who took less than 12 months of maternity leave (*N =* 288), the top five reasons reported for taking less time were: i) not wanting to significantly delay residency training, ii) rules regarding the timing of licensing exams to be able to write with their cohort, iii) financial concerns, iv) not wanting to lose clinical skills, and v) having a partner that took some parental leave. For male residents, not wanting to significantly delay residency training and having a partner that took parental leave were the two main reasons reported for not taking a longer paternity leave. Interestingly, only one third of female (*N =* 137; 31.4%) and male (*N =* 10; 30.3%) residents reported they would have liked to have taken a longer leave.

### Satisfaction with parental leave experiences

Fifty-one percent (*N =* 223) of female residents and 66.6% (*N =* 22) of male residents rated their experience of returning to residency following parental leave as either positive or very positive. Similarly, 54.7% (*N =* 239) of female residents and 63.7% (*N =* 21) of male residents reported being either satisfied or very satisfied with how well their program provided accommodations and support when they returned to residency duties. Female residents who reported being more satisfied with their program’s level of accommodation and support were more likely to report that their program director reacted positively when informed about the pregnancy (*r* =.473, *p* < .001), and more likely to report fewer challenges (*r* =.414, *p* < .001), and a greater number of accommodations offered (*r* =-.476, *p* = .000). The majority of program directors reported being either satisfied or very satisfied with how well their program provided accommodations for residents returning from maternity leave (*N =* 113; 69.2%) and residents returning from paternity leave (*N =* 116; 67.3%). Interestingly, program directors who reported having more female residents take parental leave in their program over the last 10 years reported offering more accommodations (*r* =.195, *p* = .014) and were more satisfied with the level of support and accommodation their program provided (*r* =-.226, *p* = .014), than those who reported fewer female residents taking parental leave.

### Challenges resident parents face when returning to residency following parental leave

Table 2 shows a list of common challenges residents reported during the transition period of returning to residency following parental leave. It also includes data from program directors who were asked to report what difficulties they felt resident parents face when returning from parental leave. When asked to choose which problem was the greatest, the top four choices reported by both female and male residents were: i) feeling guilty for being away from their family, ii) long and unpredictable work hours, iii) sleep deprivation, and iv) finding study time. Only 118 (27.0%) female residents and 7 (21.2%) male residents reported speaking to their program director about any struggles they were experiencing after returning from parental leave. Program directors were fairly accurate in assessing the greatest challenge for resident parents, as they felt the number one challenge for female residents is feeling guilty for being away from their family, and for male residents, sleep deprivation. For residents who wrote their licensing exam (CFPC or Royal College of Physicians and Surgeons of Canada [RCPSC]) either during or after parental leave, only 17 (3.9%) female residents and 2 (6.1%) male residents reported failing on the first try. The national failure rate for Canadian medical graduates for the 2016 CFPC exam has been reported as 6.5%,^19^ and for the RSPSC exams, averaged between 2007 and 2016, has been reported as 4.8%.^19^

**Table 2:** Challenges experienced by residents or observed by program directors when returning to residency after parental leave

Challenges	Female Residents *N* = 437	Male Residents *N* = 33	Program Directors *N* = 160
	*N* (%)	*N* (%)	*N* (%)
Feeling guilty for being away from child or family	359 (82.2%)	20 (60.6%)	121 (75.6%)
Sleep Deprivation	338 (77.3%)	27 (81.8%)	129 (80.6%)
Feeling ‘less sharp’ mentally	328 (75.1%)	12 (36.4%)	84 (52.5%)
Finding time to study for exams	324 (74.1%)	23 (69.7%)	129 (80.6%)
Feeling inadequate as a resident	324 (74.1%)	10 (30.3%)	73 (45.6%)
Feeling inadequate as a parent	314 (71.9%)	15 (45.5%)	90 (56.3%)
Long and unpredictable work hours	301 (68.9%)	23 (69.7%)	100 (62.5%)
Difficulty finding childcare or caring for sick children	190 (43.5%)	16 (48.5%)	120 (75.0%)
Decline in skills after being on leave	226 (51.7%)	6 (18.2%)	78 (48.8%)
Losing cohort of residency colleagues/study partners	210 (48.1%)	5 (15.2%)	45 (28.1%)
Difficulty with on-call responsibilities	158 (36.2%)	12 (36.4%)	83 (51.9%)
Difficulties with breastfeeding or pumping	167 (38.2%)	n/a	43 (26.9%)
Feeling burnt-out	156 (35.7%)	11 (33.3%)	29 (18.1%)
Financial stress	154 (35.2%)	16 (48.5%)	41 (25.6%)
Having to do out of town rotations	116 (26.5%)	12 (36.4%)	63 (39.4%)
Lack of role models (e.g., preceptors or colleagues with children or on leave)	94 (21.5%)	2 (6.1%)	14 (8.8%)
Lack of program-specific parental leave policies	94 (21.5%)	3 (9.1%)	8 (5.0%)
Frequent changes in workplace	90 (20.6%)	6 (18.2%)	22 (13.8%)
Lack of support or hostility from a preceptor	69 (15.8%)	1 (3.0%)	9 (5.6%)
Lack of support from extended family	67 (15.3%)	5 (15.2%)	26 (16.3%)
Lack of support or resentment from spouse	60 (13.7%)	4 (12.1%)	35 (21.9%)
Total number of challenges reported (Mean; SE)	9.52 (0.18)	6.94 (0.65)*	8.41 (0.32)

* : Does not include question about breastfeeding

A thematic analysis of the comments provided by residents and program directors allowed us to further explore these challenges in more detail. For instance, many residents commented that finding childcare was “*extremely stressful*” and they often had to rely on several options (e.g., partner, daycare/nanny, and family) to help make it work in the first year back to work, as few community daycares accept children under the age of two. Childcare appeared to be particularly difficult for surgical residents who start work early, as well as residents whose partner is a physician or resident with their own long hours and call shifts to schedule. For female residents, 110 (25.2%) reported relying on their partner for childcare, 97 (22.2%) used a community daycare, 77 (17.6%) used a nanny, 55 (12.6%) relied on a grandparent, and 52 (11.9%) used a home daycare when returning to residency. The majority of male residents relied on their partner (*N =* 20; 60.6%) or a community daycare (*N =* 5; 15.2%). Several residents stated they wished their preceptors had more experience with or education about the challenges of having children during residency. In particular, one female resident expressed what seems to be a common frustration in wishing that preceptors were better at recognizing that taking a year off for maternity leave may cause some “*mental fog*,” and some difficulty recalling information learned prior to parental leave, which might make residents appear less “*high functioning*” when they immediately return to work. However, it appears that many program directors are already aware of this issue, as one program director stated, “*Residents typically take a few weeks to get back in the groove of residency after a leave. Their performance generally remains the same or declines due to sleep deprivation, but some residents perform better because they get more organized*.”

Breastfeeding and finding time to pump at work was a major concern for many female residents. In our sample, 97.5% of female residents breastfed and the average duration was 12 months. Duration of breastfeeding was positively correlated with the length of parental leave (*r* =-.340, *p* < .001). Sixty percent of female residents continued to breastfeed or pump after returning to residency duties and 73% of these residents took less than 12 months of leave. Only 15% of female residents reported being offered breaks or a private area to breastfeed or pump. Out of 191 female residents who reported they stopped breastfeeding earlier than they had planned, 112 (58.6%) stopped because of returning to residency duties, either because it was too difficult to pump at work or because their supply permanently dropped after lengthy call shifts. Many female residents felt that residency programs should have a specific policy about pumping at work that provides basic accommodations, such as dedicated time for pumping and a private pumping space that is convenient to access, given that discrimination due to breastfeeding is a Human Rights violation in several provinces.^20^^-^^22^ Many female residents also felt there was not enough education about the benefits of breastfeeding and challenges of pumping amongst preceptors. Support for breastfeeding/pumping appeared to be particularly important for residents in surgical specialties who were less likely to take a full year of maternity leave for fear of losing their procedural skills and being ineligible to write their exam with their cohort. For instance, one female surgical resident commented, “*I took the entire year off partially so that I could facilitate breastfeeding. I would have considered returning to my residency earlier had I felt that pumping at work would have been a feasible option*.”

Finally, we also identified several new challenges when reviewing the residents’ comments. For instance, many female residents commented that a lack of communication and poor support from their preceptor during the period of transition back to residency duties after parental leave was a challenge. These residents reported wishing they had received a monthly email while on leave or at least one face-to-face meeting when returning to residency to *“check-in”* to see how they were managing, discuss their rotation schedule, assess for any challenges, and ensure they felt supported. Several male and female residents also commented on feeling stigmatized for either taking long parental leaves or attempting to improve their work-life balance for the sake of their families when returning to residency duties. For example, some female residents reported feeling pressured to take shorter parental leaves to stay competitive for fellowship programs and career advancement, to meet licensing exam eligibility requirements, to keep up their clinical skills, or to appease fellow residents who may have to cover their absence. Finally, several male residents reported feeling pressured to work long hours after returning from parental leave in order to appear dedicated to their training program (e.g., one male resident stated, “*My desire to be available for my child and family was seen as a lack of interest in being at work*”).

### Accommodations offered to resident parents by program directors

Table 3 shows a list of common accommodations offered to resident parents and includes the percentage of residents in our study who reported being offered these accommodations, and the percentage of program directors who reported offering these accommodations.

**Table 3:** Accommodations offered to residents by program directors when returning to residency after parental leave

Accommodations	Female Residents (*N* = 437)	Male Residents (*N* = 33)	Program Directors (*N* = 159)
	*N* (%)	*N* (%)	*N* (%)
Using vacation time before or after leave	157 (35.9%)	22 (66.7%)	115 (72.3%)
Flexibility in rotation schedule	123 (28.1%)	14 (42.4%)	125 (78.6%)
Leaving early or day off when child is sick or for appointments	107 (24.5%)	15 (45.5%)	118 (74.2%)
Doing academic work during leave	100 (22.9%)	4 (12.1%)	76 (47.8%)
Avoiding out-of-town rotations (or help with accommodations)	91 (20.8%)	5 (15.2%)	89 (56.0%)
Option to extend parental leave	79 (18.1%)	12 (36.4%)	97 (61.0%)
Breaks to breastfeed or pump	68 (15.6%)	n/a	68 (42.8%)
Private area to breastfeed or pump	65 (14.9%)	n/a	63 (39.6%)
Refrigerated storage for pumped breastmilk	57 (13.0%)	n/a	62 (39.0%)
Returning part-time or with modified hours	52 (11.9%)	3 (9.1%)	49 (30.8%)
Dedicated time to study for exams	48 (11.0%)	4 (12.1%)	51 (32.1%)
Having a mentor/role-model for support	44 (10.1%)	3 (9.1%)	56 (35.2%)
Handbook for resident parents	8 (1.8%)	0	3 (1.9%)
**Total number of accommodations reported (Mean; SE)**	**2.28 (0.97)**	2.82 (0.36)*	**6.11 (0.23)**

* : Does not include questions about breastfeeding

**Figure 1:**
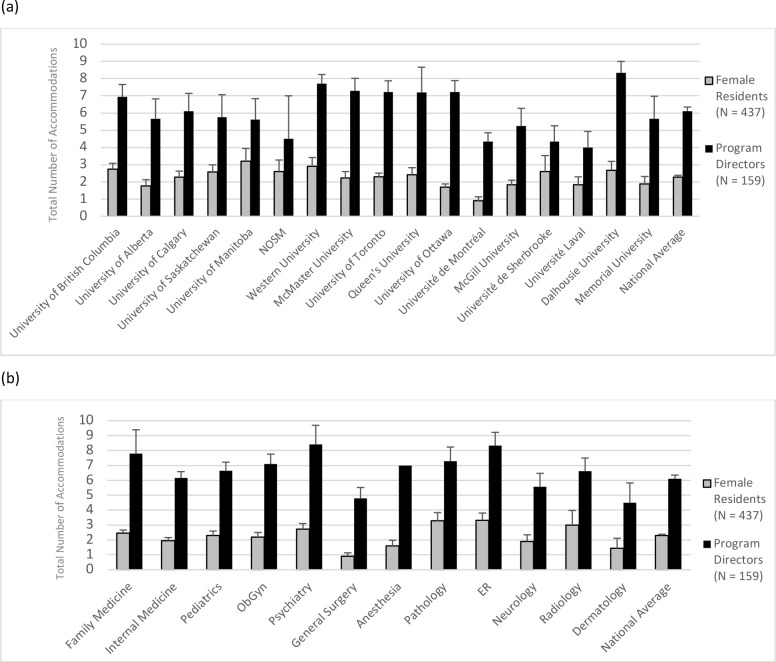
Total mean number of accommodations (with standard errors) offered to resident parents (a) across all Canadian medical schools and (b) across the most common residency programs

Figure 1 illustrates the total mean number of accommodations offered to resident parents across the 17 Canadian medical schools and across the most common residency programs, from both the female resident and program director perspectives. Although there are many factors to consider when making comparisons across schools or residency programs, the data in these figures illustrate not only the high degree of variability in accommodations provided by program directors across medical schools and certain residency programs, but also the significant mismatch between female resident and program director perspectives. Direct comparisons between female residents and program directors from the same school and program are discussed below. Comments from programs directors also highlighted the significant variability in the degree of support and accommodation provided to resident parents when transitioning back to work. For instance, one program director stated:

*I think it's very important to have an understanding of the stress that parents face when they return to residency training after maternity or paternity leave. I wish there was more transparency or direction from our PGME office about options or accommodations that can be offered to these parents*.

While another program director commented,

*Accommodations are made in the form of the leave itself. I do not see a good reason to make more accommodations. It is a choice to have children so when residents are back at work, they should be back at work (to do otherwise is unfair to those who choose not to have children)*.

When residents were given the option to comment on other possible accommodations that might be helpful, many suggested having a re-acclimatization period in the first month of returning to residency, with refresher courses or a gradual increase in hours and call shifts. One resident stated, “*I think the very first month after maternity leave is crucial as it’s a transition phase in terms of learning and juggling work-life balance*.” Many female residents wished they were given protected study time during weekdays, especially during the year of their licensing exam, as it can be challenging to find time to study outside work hours with a young child at home. Some residents report using their vacation time to study, while others proposed either extending residency to allow for a dedicated study block, or allowing residents to attend academic/teaching sessions during their parental leave so that they can use this time slot for studying when they return from parental leave. Male residents uniquely requested longer paid leave, and reduced stigma.

Both female residents and program directors felt that it was important for universities to provide clear program-specific policies and guidance for parental leave. In particular, residents requested knowing which accommodations were available, such as whether part-time return or waivers of training might be possible. Waivers of training, which are typically a maximum of three months for RCPSC residents and one month for family medicine residents, were of particular interest to the male residents in our study, who did not want to significantly delay their training. Several residents noted they could have used practical advice on work-life balance, knowing what memberships can be suspended and re-started after parental leave (e.g., CMPA), accrued vacation weeks while on leave, and information regarding the rules for being eligible to write their licensing exam or for taking a second parental leave during residency. Only eight (*N =* 1.8%) female residents reported receiving a handbook with information for resident parents.

### Comparison between female residents and program directors

Due to unequal sample sizes between the female resident and program director groups, direct comparisons between these two groups were done using t-tests after matching each program director to a female resident from the same school and same program (all 17 medical schools were represented in 99 matched pairs). When data from more than one female resident or program director from a particular school and program were available, random sampling in SPSS was used to choose matched pairs. Levene's test was calculated to ensure homogeneity of variance for the t-tests. In cases where Levene's test was significant, (i.e., heterogeneity of variance in the two groups), adjusted p-values are reported, while the original degrees of freedom (*DF*) are reported.

There was no significant difference in the total number of challenges reported by female residents and program directors, when the groups were matched to both school and program. However, there was a statistically significant difference in the total number of accommodations reported by matched female residents and program directors, whereby program directors reported offering significantly more accommodations than female residents reported being offered, *t(196)* = 13.06, *p* < .001, Cohen’s *d* = 1.86 (see Figure 1). A comment from a female resident in this study may help explain this finding:

*I was able to advocate for several accommodations, but none were offered to me. I think this is an important issue, the processes were not standardized or transparent at all. I realize now that people will generally be supportive to new parents but you have to communicate your needs, which I was a little nervous of doing for fear of sounding lazy or weak*.

Finally, when female residents and program directors were compared using matched samples, program directors reported being significantly more satisfied with the accommodations and support provided by their program than female residents, *t(200)* = 3.53, *p* = .001, Cohen’s *d* = 0.50).

### Change in female residents’ experiences over time

In order to examine the change in female residents’ experiences over time, correlational analyses were conducted using the year residents started residency and other variables of interest. There was a significant correlation between residency year and residency program (e.g., Family Medicine versus all RCPSC specialty programs), *r* =-.180, p=.002, with more recent graduates being from family medicine. Thus, for each significant correlation, follow-up analyses were conducted separately for female residents in family medicine (N =121) and female residents in RCPSC specialty programs (*N =* 314). Female residents who started their residency program more recently reported receiving more positive reactions from their program directors when they were informed of the pregnancy than those who started residency less recently (*r* =-.180, *p* < .001). Further analyses revealed this finding was significant for RCPSC residents (*r* =-.176, p=.002), but not significant for family medicine residents. Residents who started more recently also reported having more challenges (*r* = .132, *p* = .007); however, this finding was significant for RCPSC residents (*r* = .178, *p* = .002), but not for family medicine residents. Finally, residents who started more recently reported being offered more accommodations than their predecessors (*r* = .133, *p* = .006); however, this finding was significant for family medicine residents (*r* = .246, *p* = .006), but not for RCPSC residents.

## Discussion

Our study used one of the largest and most heterogeneous samples of Canadian resident parents and program directors to gather important national data on the experiences of resident parents as they return to residency after parental leave. Our study is also one of the first to examine which accommodations are currently being offered to resident parents from both the resident and program director perspectives. Our findings indicate that program directors appear to be aware of the most common challenges resident parents face. They reported offering significantly more accommodations than female residents in the same residency program at the same institution report being offered. One possible explanation of these results could be that program directors might only offer these accommodations *to certain residents if they are asked on a case-by-case basis*, as many residents in the present study stated they were unaware that they could ask for accommodations and did not speak to their program directors when challenges arose. Given than most resident parents, regardless of their residency program or institution, face the same types of challenges when they return to residency (e.g., childcare, sleep deprivation, breastfeeding, study time, etc.), it seems only fair that all resident parents should be offered the same accommodations. However, our findings, as illustrated in Figure 1, suggest there is considerable variability in the level of support provided by program directors to resident parents across different schools and different programs. Clearly, there is a need for better communication between resident parents and program directors, as well as greater awareness of the existing parental leave policies within specific residency programs at each institution.

Residents should be encouraged to inform their program director about their need for parental leave as soon as possible to allow for more flexibility in rotation scheduling and less disruption to the workload of their peers. Our data show that most program directors react positively when being informed about the need for parental leave, especially when they are informed earlier in the pregnancy. Given that some female residents may be hesitant to reveal their pregnancy until the risk of miscarriage is lower,^4^ program directors can encourage early disclosure by ensuring this information is kept confidential, offering support regardless of the pregnancy outcome, and informing residents that it is in their best interest to disclose as early as possible. Based on feedback received from residents in this study, we recommend that program directors meet face-to-face with residents planning to take a parental leave at least once, and continue to have regular communication in-person or over email when the resident returns after leave to provide additional support. These sessions could help program directors identify the most critical challenges and most necessary accommodations for their own specific program, which could aid in the development of better parental leave polices.

Fortunately, our findings suggest that support for resident parents is improving, as female residents who started their residency more recently reported receiving a more positive reaction from their program director when informing them of their pregnancy, and for family medicine residents, being offered more accommodations. Interestingly, RCPSC residents who started their training more recently reported having more challenges than their predecessors. Perhaps this finding reflects a shift in culture and higher expectations of accommodations, with more resident mothers attempting to breastfeed and pump at work, as well as attempting to avoid burnout and improve their work-life balance.

Despite numerous articles highlighting the need for written program-specific parental leave policies, few residency programs appear to have one in place, especially for the transition period when residents return to duties following parental leave.^11^^,^^18^ The lack of clear policies and guidelines for accommodating resident parents as they return to work results in substantial variability in the number of accommodations offered by program directors in the same residency program or at the same institution. This can be frustrating for both resident parents, and program directors. Not surprisingly, the most supportive program directors in our study were those who had the most experience dealing with residents taking parental leave or had a child themselves during residency, making them more aware of the challenges their residents face.

Ideally, all residency programs would have their own written parental leave policy that: i) includes information about which accommodations might be possible for resident parents on a case-by-case basis (e.g., waivers of training, part-time return to work, accrued vacation weeks while on leave), ii) adheres to federal law and the provincial resident union contract, iii) is inclusive for all types of parents (e.g., adoption), and iv) is accessible to all residents. Prospective residents may be particularly interested in this information as it could affect their decision about where to do their residency training.

At a minimum, all residency programs should ensure they provide adequate support for breastfeeding mothers through the provision of adequate time and a private space for pumping breast milk, as this is a human rights requirement. Providing breastfeeding support for resident mothers may enable some residents to return to residency earlier or return part-time, which might be important for those who want to keep their clinical and procedural skills proficient. All residents, regardless of whether they plan to have a child or not, should be made aware of online resources for resident parents. For instance, comprehensive handbooks and checklists for residents planning to take parental leave during residency have recently been created and made available online by a few Canadian residency programs^23,^^24^ and resident associations^16,^^25^ Finally, our study identifies several topics, such as the benefits of breastfeeding, the challenges of pumping at work, and the cognitive fatigue in the first few weeks back at work, that could be topics for faculty development for preceptors and program directors.

This study has several limitations. First, we were unable to collect data from all program directors or all residents who took parental leave in the last 20 years; thus, our recruitment of participants may suffer from selection bias. It is possible that residents with more negative experiences or program directors who are more supportive of resident parents may have been more interested in participating in our study. Similarly, because we relied on self-reports from residents who completed their training over the last 20 years, it is possible that not all residents’ experiences were recalled accurately or completely, leading to recall bias in our data. Given that we were unable to collect data from all program directors across Canada, unequal sample sizes across schools and programs limited our ability to make any direct comparisons across these demographic variables. Next, we did not collect data on the sex of the program directors, the number of years they had served as program director, or whether they had a child during their own residency training, which may have been interesting to examine in terms of the level of support they provide resident parents. Finally, when using resident-program director pairs matched by school and program, we were unable to match by year, which may have influenced our results. For instance, our participants included *current* program directors, whereas our sample of female residents included those who started residency between 1998 and 2018 (mean year of starting residency = 2011). If current program directors offer more accommodations to resident parents than their predecessors, this could explain the significant difference we found between program directors and female residents in the total number of accommodations offered. However, when we excluded matched resident-program director pairs where the female resident started residency earlier than 2013, there was still a significant group difference *t(88)* = 9.43, *p* < .001, where program directors reported offering more accommodations than female residents reported being offered.

Our study demonstrates that there are still significant improvements to be made in supporting resident parents in Canada as they return from parental leave. On a positive note, our study shows that many program directors are aware of the challenges that resident parents face after taking parental leave and are willing to discuss possible accommodations on a case-by-case basis. Many residents offered possible solutions that could have reduced the challenges they experienced and improved their work-life balance during the transition back to residency training. While it may not be possible logistically to provide resident parents with all their requested accommodations, ensuring all resident parents have access to basic accommodations (e.g. breastfeeding support), regular contact and support from their program director, and a written, detailed, program-specific parental leave policy at the start of their training, could considerably enhance the experiences of both resident parents and program directors as they navigate through this challenging transition period.

## Footnotes

1 Estimated total number of maternity/paternity leaves in the last 10 years nationwide = sum of the mean number of maternity/paternity leaves in last 10 years per program as reported by program directors in our study x number of medical schools with that program.

## Appendix A

**Table 1:** Demographic data

Variable	Female Residents (N = 437)	Male Residents (N = 33)	Program Directors (N =172)
***Mean Age at Delivery****(Range)*	30.79 (23-42)	31.06 (28-41)	n/a
***Mean Year Starting Residency****(Range)*	2011 (1998-2018*)	2013 (2005-2018)	n/a
***Postgraduate Year of Starting Parental Leave*** PGY1 PGY2 PGY3 PGY4 PGY5 PGY6/Fellowship Missing	***N (%)*** 75 (17.2%) 130 (29.7%) 96 (22.0%) 71 (16.2%) 56 (12.8%) 6 (1.4%) 3 (0.7%)	***N (%)*** 8 (24.2%) 9 (27.3%) 8 (24.2%) 5 (15.2%) 2 (6.1%) 0 1 (3.0%)	n/a
***Medical School****:* University of British Columbia University of Alberta University of Calgary University of Saskatchewan University of Manitoba NOSM± Western University McMaster University University of Toronto Queens University University of Ottawa McGill University Université de Montréal Université de Sherbrooke Université Laval Dalhousie University Memorial University Missing	***N (%)*** 47 (10.8%) 22 (5.0%) 42 (9.6%) 21 (4.8%) 20 (4.6%) 10 (2.3%) 20 (4.6%) 31 (7.1%) 70 (16.0%) 24 (5.5%) 38 (8.7%) 23 (5.3%) 10 (2.3%) 5 (1.1%) 12 (2.7%) 24 (5.5%) 16 (3.7%) 2 (0.5%)	***N (%)*** 2 (6.1%) 0 8 (24.2%) 1 (3.0%) 1 (3.0%) 0 3 (9.1%) 5 (15.2%) 0 0 4 (12.1%) 2 (6.1%) 1 (3.0%) 0 2 (6.1%) 4 (12.1%) 0 0	***N (%)*** 17 (9.9%) 9 (5.2%) 10 (5.8%) 4 (2.3%) 8 (4.7%) 2 (1.2%) 7 (4.1%) 15 (8.7%) 19 (11.0%) 6 (3.5%) 9 (5.2%) 14 (8.1%) 15 (8.7%) 11 (6.4%) 12 (7.0%) 7 (4.1%) 7 (4.1%) 0
***Residency Program****:* Anatomical Pathology Anesthesiology Dermatology Diagnostic Radiology Emergency Medicine Family Medicine (2-year program) Family Medicine with an Enhanced Skill General Pathology General Surgery Hematological Pathology Internal Medicine Internal Medicine Sub-Specialty Medical Genetics Medical Microbiology Neurology Neurology – Pediatric Neuropathology Neurosurgery Obstetrics and Gynecology Ophthalmology Orthopedic Surgery Otolaryngology Pediatrics Pediatrics – Clinical Investigator Pediatrics Sub-Specialty Physical Medicine & Rehabilitation Plastic Surgery Psychiatry Public Health & Preventative Medicine Radiation Oncology Urology Missing	***N (%)*** 13 (3.0%) 22 (5.0%) 7 (1.6%) 6 (1.4%) 16 (3.7%) 116 (26.5%) 4 (0.9%) 2 (0.5%) 18 (4.1%) 3 (0.7%) 39 (8.9%) 29 (6.6%) 2 (0.5%) 3 (0.7%) 11 (2.5%) 2 (0.5%) 0 1 (0.2%) 39 (8.9%) 3 (0.7%) 1 (0.2%) 0 31 (7.1%) 1 (0.2%) 8 (1.8%) 4 (0.9%) 3 (0.7%) 36 (8.2%) 5 (1.1%) 4 (0.9%) 1 (0.2%) 7 (1.6%)	***N (%)*** 1 (3.0%) 3 (9.1%) 0 2 (6.1%) 7 (21.2%) 6 (18.2%) 0 0 0 0 5 (15.2%) 0 0 1 (3.0%) 1 (3.0%) 0 0 2 (6.1%) 0 1 (3.0%) 0 1 (3.0%) 0 0 0 0 0 3 (9.1%) 0 0 0 0	***N (%)*** 4 (2.3%) 1 (0.6%) 4 (2.3%) 8 (4.7%) 3 (1.7%) 5 (2.9%) 4 (2.3%) 1 (0.6%) 6 (3.5%) 0 7 (4.1%) 45 (26.2%) 1 (0.6%) 0 7 (4.1%) 1 (0.6%) 2 (1.2%) 4 (2.3%) 18 (10.5%) 4 (2.3%) 6 (3.5%) 1 (0.6%) 6 (3.5%) 0 10 (5.8%) 5 (2.9%) 2 (1.2%) 6 (3.5%) 2 (1.2%) 3 (1.7%) 2 (1.2%) 4 (2.3%)
***Number of children delivered during residency*** 1 2 3 Twins	***N (%)*** 314 (71.9%) 101 (23.1%) 8 (1.85%) 14 (3.2%)	***N (%)*** 20 (60.6%) 11 (33.3%) 1 (3.0%) 1 (3.0%)	n/a
***Birth order of first child born during residency*** First-born Second-born Third-born Fourth-born Twins	***N (%)*** 391 (89.5%) 28 (6.4%) 4 (0.9%) 2 (0.5%) 12 (2.7%)	***N (%)*** 26 (78.8%) 4 (12.1%) 0 2 (6.1%) 1 (3.0%)	n/a
***Marital Status at delivery:*** Married Common-Law Separated or Divorced Single	***N (%)*** 392 (89.7%) 42 (9.6%) 1 (0.2%) 2 (0.5%)	***N (%)*** 30 (90.9%) 3 (9.1%) 0 (0%) 0 (0%)	n/a
***Partner was a physician, resident or medical student***	***N (%)*** Yes: 81 (18.6%) No: 355 (81.2%) Missing: 1 (0.2%)	***N (%)*** Yes: 13 (39.4%) No: 16 (48.5%) Missing: 4 (12.1%)	n/a

Note: *The one Resident who started their program in 2018 took a 2-month maternity leave; ±NOSM = Northern Ontario School of Medicine
